# Extracorporeal membrane oxygenation (ECMO) and the acute respiratory distress syndrome (ARDS): a systematic review of pre-clinical models

**DOI:** 10.1186/s40635-019-0232-7

**Published:** 2019-03-25

**Authors:** Jonathan E. Millar, Nicole Bartnikowski, Viktor von Bahr, Maximilian V. Malfertheiner, Nchafatso G. Obonyo, Mirko Belliato, Jacky Y. Suen, Alain Combes, Daniel F. McAuley, Roberto Lorusso, John F. Fraser, on behalf of the European Extracorporeal Life Support Organisation (EuroELSO) Innovations Workgroup, the National Health Medical Research Council Australia Centre of Research Excellence for Advanced Cardio-respiratory Therapies Improving Organ Support (NHMRC CREACTIONS)

**Affiliations:** 1 Critical Care Research Group, The Prince Charles Hospital, Brisbane, 4035 QLD Australia; 2 Faculty of Medicine, University of Queensland, Brisbane, Australia; 3 School of Chemistry, Physics and Mechanical Engineering, Queensland University of Technology, Brisbane, Australia; 4 Department of Physiology and Pharmacology, Section for Anesthesiology and Intensive Care Medicine, Karolinska Institutet, Stockholm, Sweden; 5 Department of Internal Medicine II, Cardiology and Pneumology, University Medical Center Regensburg, Regensburg, Germany; 6 Wellcome Trust Centre for Global Health Research, Imperial College London, London, UK; 7 U.O.C. Anestesia e Rianimazione 1, IRCCS, Policlinico San Matteo Foundation, Pavia, Italy; 8 Medical-Surgical Intensive Care Unit, Hôpital Pitié-Salpêtrière, Assistance Publique-Hôpitaux de Paris, Paris, France; 9 Institute of Cardiometabolism and Nutrition, Sorbonne University, Paris, France; 10 Wellcome-Wolfson Centre for Experimental Medicine, Queen’s University Belfast, Belfast, UK; 11 Department of Cardiothoracic Surgery, Heart & Vascular Centre, Maastricht University Medical Hospital, Maastricht, Netherlands

**Keywords:** Extracorporeal membrane oxygenation, Acute respiratory distress syndrome, Animal models, Pre-clinical models, Systematic review

## Abstract

**Objectives:**

Extracorporeal membrane oxygenation (ECMO) is an increasingly accepted means of supporting those with severe acute respiratory distress syndrome (ARDS). Given the high mortality associated with ARDS, numerous animal models have been developed to support translational research. Where ARDS is combined with ECMO, models are less well characterized. Therefore, we conducted a systematic literature review of animal models combining features of experimental ARDS with ECMO to better understand this situation.

**Data sources:**

MEDLINE and Embase were searched between January 1996 and December 2018.

**Study selection:**

Inclusion criteria: animal models combining features of experimental ARDS with ECMO. Exclusion criteria: clinical studies, abstracts, studies in which the model of ARDS and ECMO has been reported previously, and studies not employing veno-venous, veno-arterial, or central ECMO.

**Data extraction:**

Data were extracted to fully characterize models. Variables related to four key features: (1) study design, (2) animals and their peri-experimental care, (3) models of ARDS and mechanical ventilation, and (4) ECMO and its intra-experimental management.

**Data synthesis:**

Seventeen models of ARDS and ECMO were identified. Twelve were published after 2009. All were performed in large animals, the majority (*n* = 10) in pigs. The median number of animals included in each study was 17 (12–24), with a median study duration of 8 h (5–24). Oleic acid infusion was the commonest means of inducing ARDS. Most models employed peripheral veno-venous ECMO (*n* = 12). The reporting of supportive measures and the practice of mechanical ventilation were highly variable. Descriptions of ECMO equipment and its management were more complete.

**Conclusion:**

A limited number of models combine the features of experimental ARDS with ECMO. Among those that do, there is significant heterogeneity in both design and reporting. There is a need to standardize the reporting of pre-clinical studies in this area and to develop best practice in their design.

**Electronic supplementary material:**

The online version of this article (10.1186/s40635-019-0232-7) contains supplementary material, which is available to authorized users.

## Introduction

In recent years, the use of extracorporeal membrane oxygenation in patients with acute respiratory distress syndrome (ARDS) has grown substantially [1]. ECMO is now an accepted technique for temporarily supporting those with severe ARDS whose condition is refractory to conventional management [2, 3]. Despite advances in our understanding of the pathophysiology of ARDS, mortality among patients remains high, with only a modest improvement over the last decade [4]. A contributing factor may be the failure to successfully translate a proven therapeutic strategy for the treatment of ARDS [5]. Substantial effort has been devoted to this endeavor, and correspondingly numerous animal models of ARDS have been developed to assist in the investigation and translation of novel interventions [6]. As the use of ECMO in ARDS matures, it will become increasingly important to evaluate candidate ARDS therapies in the unique context of extracorporeal circulation [7]. Likewise, interventions primarily associated with ECMO require established pre-clinical models to facilitate progress toward clinical trials. There are fewer well-characterized models which combine experimental ARDS with ECMO than ARDS alone. To better understand existing animal models of ARDS and ECMO, we have undertaken a systematic review of studies reporting novel models in animals. A systematic appreciation of animal models which include the use of ECMO will allow us to identify current limitations, establish areas for innovation and improvement, and will assist in the creation of a minimum data set for pre-clinical ECMO studies.

## Materials and methods

### Design

A systematic review protocol was constructed in advance and published on the Systematic Review Centre for Laboratory Animal Experimentation (SYRCLE) website (https://issuu.com/radboudumc/docs/animal_models_of_acute_respiratory_?e=28355229/48256411). The protocol addresses the requirements of the Preferred Reporting Items for Systematic Review and Meta-analysis Protocols (PRISMA-P) statement [8]. The published protocol was amended after publication to remove the requirement for papers to be published in English. A native language speaker was identified to translate those not appearing in English.

### Search strategy

We searched the MEDLINE (via PubMed) and Embase (via Ovid SP) indexed online databases from January 1996 to December 2018. The search strategy was designed in conjunction with a trained medical librarian (see Additional file 1 for the full search strategy). The filters used to identify animal studies were those previously described and validated by de Vries et al. [9] and Hooijmans et al. [10]. Citations were collected in a reference management software program (EndNote™, Clarivate Analytics, PA, USA).

### Study selection

Study selection occurred in two phases. Firstly, abstracts and citations were independently screened for relevance by two authors (JM and NB). Discrepancies were resolved by reference to a third author (MM). Articles were excluded on the following basis: (1) if they were not performed in animals, (2) if they did not involve the use of ECMO, or (3) if they did not include a model of ARDS. The full text of articles deemed relevant was retrieved. There were no language restrictions. Articles not published in English were translated by a native speaker. In the second phase, full-text articles were independently reviewed (JM, NB) and excluded if (1) they did not report an animal model, (2) they did not use veno-venous, veno-arterial, or central ECMO, (3) they did not include a model of ARDS, (4) they were in abstract format, or (5) if the same model of ARDS and ECMO had been reported in a previous publication. Disagreements were resolved by a third author (MM). The reference lists of screened studies were reviewed to identify publications not found by the original search strategy.

### Study characteristics and data abstraction

Included studies were jointly reviewed by JM, NB, and VB. Data were extracted using a pre-piloted data extraction form (Additional file 2). Disagreements were resolved by reference to a senior member of the team. Descriptive data for each study were abstracted including the title, author(s), year of publication, and journal title. Detailed data were identified in relation to four major categories:

1. *Study design*. The aim(s) and hypothesis of the study was recorded, as were elements related to study design, such as randomization procedures, blinding, the use of sub-groups, and sample size.
2. *Animals and their peri-experimental care*. This included information on the species, strain, age, weight, and gender of the animals used in experiments. Additional data were abstracted on anesthesia, monitoring, fluid management, intra-experimental drug administration, and euthanasia.
3. *Models of ARDS and mechanical ventilation*. Details were extracted on the means of inducing experimental ARDS and on the definition of ARDS applied in each study. Additional data were extracted to assess mechanical ventilation practices before and during ECMO.
4. *Models of ECMO and its intra-experimental management*. Data were recorded on the mode of ECMO employed, devices used, the method and configuration of cannulation, priming, flow rates, pump speeds, sweep gas settings, anticoagulation practices, and the duration of extracorporeal support.

Studies published after 2011 were assessed for compliance with the Animal Research: Reporting of In Vivo Experiments (ARRIVE) guidelines [11].

### Data synthesis and analysis

Data were tabulated for ease of comparison. Summary statistics were used as appropriate. Given the heterogeneous nature of included studies and the aim of this review, to characterize and assess the quality of the models rather than the study outcomes, no attempt was made at meta-analysis.

## Results

**Fig. 1:**
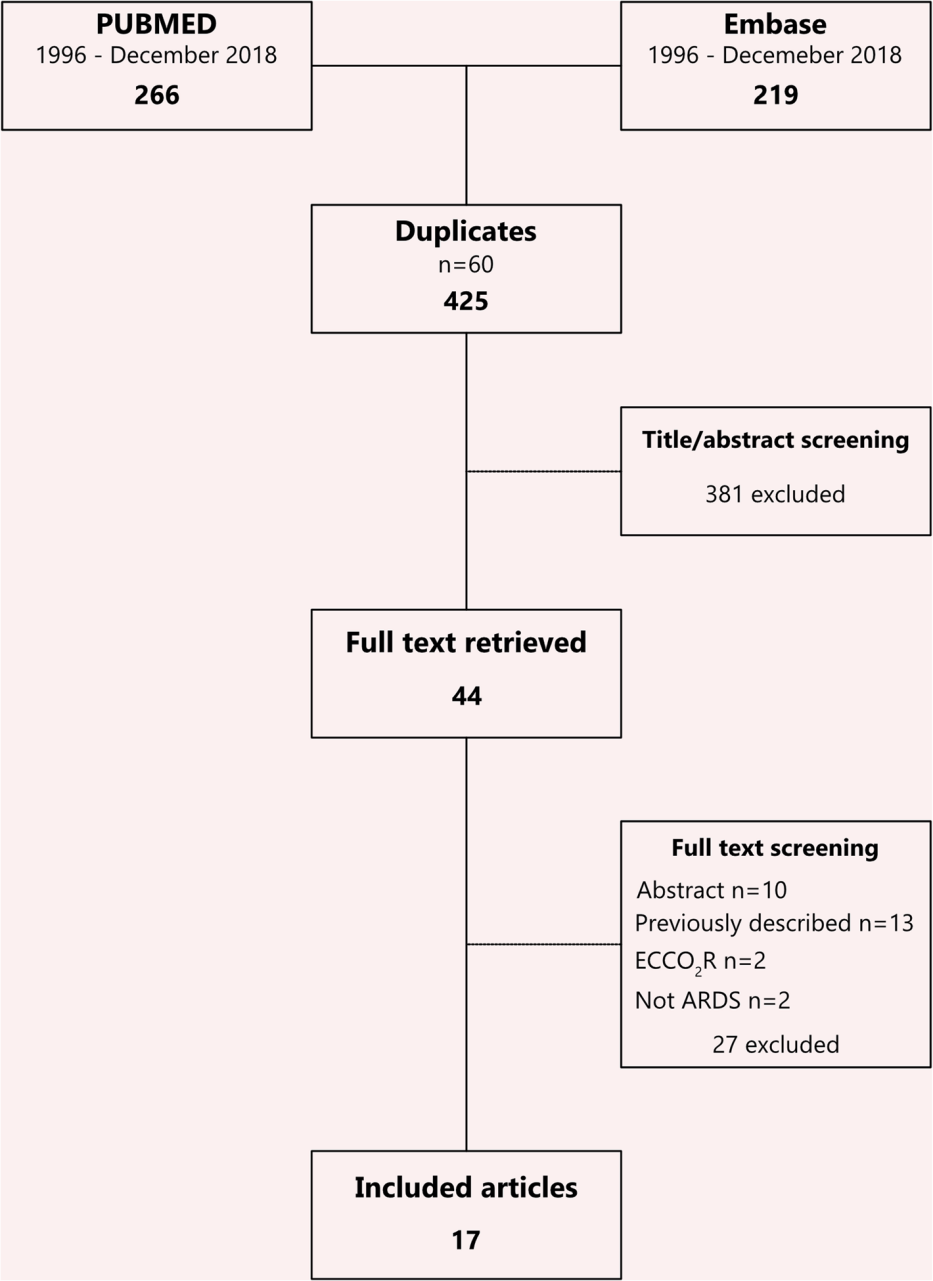
PRSIMA flow diagram for inclusion and exclusion criteria

A total of 370 unique citations were identified in our search. Of these, 44 passed the first phase of screening and had full-text articles retrieved. After secondary screening, 17 articles met the inclusion criteria and were included in the final analysis [12–28]. Figure 1 shows the PRISMA flow diagram for study inclusion and exclusion.

### Description of included studies

An overview of included studies is included in Table 1. More than two thirds (*n* = 12) were published after 2009. Most studies were conducted in Europe (*n* = 5) and Asia (*n* = 5), followed by North America (*n* = 4), South America (*n* = 2), and Australasia (*n* = 1). The purpose of studies varied but included physiological studies (*n* = 6), technology evaluations (*n* = 5), and interventional trials (*n* = 5). All studies were conducted in large animals, the majority in pigs (*n* = 10), followed by sheep (n = 6) and a single canine study. No small animal models met the inclusion criteria. The median number of animals studied was 17 (12–24), with the largest two studies using 30 animals. The median duration of included studies was 8 (5–24) hours, with two studies, both in pigs, reporting recovery and follow-up of 7 and 14 days, respectively [26].

**Table 1:** Description of studies included in the systematic review

Study	Year	Species	Study type	Number	ARDS model type	ECMO configuration	Study duration (hours)
Kim et al. [18]	2004	Dog	Technology evaluation	16	Oleic acid	VA (central)	2
Araos et al. [12]	2016	Pig	Model development	18	Saline lavage + injurious ventilation	VV	24
Wang et al. [28]	2016	Pig	Interventional	28	LPS infusion	VV	24 + 14-day recovery
Ni et al. [23]	2015	Pig	Physiological	30	Blunt injury	VV	24
Pilarczyk et al. [25]	2015	Pig	Technology evaluation	14	Saline lavage	VV	8
Park et al. [24]	2013	Pig	Physiological	5	Saline lavage + fecal peritonitis	VV	Unclear
Kopp et al. [20]	2011	Pig	Technology evaluation	6	Hypoxia	VV	4
Song et al. [26]	2010	Pig	Interventional	28	LPS infusion	VV	24 + 7-day recovery
Kopp et al. [21]	2010	Pig	Technology evaluation	24	Saline lavage	VV	24
Henderson et al. [16]	2004	Pig	Interventional	24	Oleic acid	VA	8
Dembinski et al. [13]	2003	Pig	Technology evaluation	12	Saline lavage	VV	6
Kocyildrim et al. [19]	2017	Sheep	Interventional	11	LPS infusion	VV	4
Hou et al. [17]	2015	Sheep	Physiological	20	Hypoxia	VA (central)	unclear
Langer et al. [22]	2014	Sheep	Physiological	11	Oleic acid	VV	22
Shekar et al. [14]	2012	Sheep	Physiological	17	Smoke inhalation	VV	2–24
Totapally et al. [27]	2004	Sheep	Physiological	17	Saline lavage + HCL acid instillation	VA	6
Germann et al. [15]	1997	Sheep	Interventional	30	Oleic acid	VV (central)	5

*LPS* lipopolysaccharide, *HCL* hydrochloric acid, *VA* veno-arterial, *VV* veno-venous

### Animals and their peri-experimental care

A summary of the peri-experimental care of animals is provided in Table 2. More than half (*n* = 9) of studies used exclusively female animals, while five did not report gender. The age of the animals was inconsistently documented, with 10 studies omitting this detail. The majority of investigators used total intravenous anesthesia (*n* = 14), including the two studies that involved recovery from anesthesia [26, 28]. Ketamine was the most commonly used anesthetic, with a maintenance dose range between 5 and 10 mg/kg/h. Inhalational anesthesia was used in two studies [16, 19]. Only four studies reported a protocolized approach to cardiovascular support [12, 24, 25, 27], while six studies provided data on cumulative fluid balance.

**Table 2:** Details of anesthetic, airway, and fluid management

Study	Age	Weight (kg)	Gender	Airway	Anesthesia		Paralysis	Fluid therapy		
					Induction	Maintenance		Type	Rate/volume	
Dogs										
Kim et al. [18]		20–25								
Pigs										
Araos et al. [12]		30 ± 5		ETT	Ketamine, midazolam, fentanyl	Ketamine, midazolam, fentanyl	Atricurium	Crystalloid	2 mL/kg/h	
Wang et al. [28]	4–6 weeks	7–8	F	ETT	Ketamine, diazepam	Ketamine, diazepam				
Ni et al. [23]	Juvenile	30 ± 5	M + F	Trach	Ketamine, diazepam	Ketamine, diazepam		Crystalloid	3 mL/kg/h	
Pilarczyk et al. [25]		57–62	F	ETT	Ketamine, azaperone	Propofol, midazolam, fentanyl		Crystalloid	3 mL/kg/h	
Park et al. [24]		79–81	F		Thiopentone	Midazolam, fentanyl	Pancuronium	Crystalloid	3 mL/kg/h	
Kopp et al. [20]		37 ± 1	F	ETT	Ketamine, thiopentone, azaperone	Thiopentone, fentanyl		Crystalloid + HES		
Song et al. [26]	4–5 weeks	9–14	M	ETT	Ketamine	Ketamine, fentanyl		Crystalloid		
Kopp et al. [21]		45 ± 6	F	ETT	Ketamine, thiopentone, azaperone	Thiopentone, fentanyl		Crystalloid + HES		
Henderson et al. [16]	Juvenile	7.7–15.0		ETT	Isoflurane	Isoflurane, fentanyl		Crystalloid		
Dembinski et al. [13]		37 ± 3	F	ETT	Thiopentone, ketamine, azaperone	Thiopentone, fentanyl		HES		
Sheep										
Kocyildrim et al. [19]		36.5–65		ETT	Ketamine	Isoflurane		Crystalloid	1 mL/kg/h	
Hou et al. [17]	2 years	40 ± 5	M	ETT	Propofol	Sufentanil	Atricurium			
Langer et al. [22]		45 ± 6	F	Trach	Isoflurane, tiletamine-zolazepam, buprenorphine	Midazolam, buprenorphine		Crystalloid	150–200 mL/h	
Shekar et al. [14]	1–3 years	4–50	F	Trach	Alfaxalone, midazolam	Ketamine, alfaxalone, midazolam, buprenorphine		Crystalloid	2 mL/kg/h	
Totapally et al. [27]	2–6 weeks	3.6–12.7		Trach	Ketamine	Ketamine	Vecuronium	Crystalloid	5 mL/kg/h	
Germann et al. [15]		35–40	F			Thiopentone				

*ETT* endotracheal tube, *Trach* tracheostomy, *HES* hydroxyethyl starch

### Models of ARDS and mechanical ventilation

A summary of the means of inducing experimental ARDS in studies is contained in Table 1. A range of ARDS models are described, including oleic acid (OA) infusion (*n* = 4), lipopolysaccharide (LPS) infusion (*n* = 3), saline lavage (*n* = 3), hypoxia (*n* = 2), blunt injury (*n* = 1), and smoke inhalation (*n* = 1). Three further studies combined saline lavage with a secondary injury. Definitions of experimental ARDS were varied and not universally reported (Table 3). Likewise, mechanical ventilatory practices, both before and during ECMO, were incompletely described (Table 4).

**Table 3:** Detailed methods of inducing experimental ARDS and definitions of injury

Study	Detailed injury methods	Definition of injury (experimental ARDS)
Kim et al. [18]	0.1 mL/kg i.v. OA over 30 min	P/F < 150 mmHg
Araos et al. [12]	Saline lavage (30 mL/kg at 39 °C) × 4 (2 prone, 2 supine) and 2 h of injurious ventilation (inspiratory pressure 40 cmH_2_O, PEEP 0 cmH_2_O, FiO_2_ 1.0, RR 10)	P/F < 250 mmHg
Wang et al. [28]	18–20 μg/kg i.v. *E. coli* LPS within 1 h	P/F ≤ 300 mmHg and 30% decrease in dynamic compliance from baseline
Ni et al. [23]	Blunt injury (free fall 0.45 kg weight from 1-m column) to each lateral chest wall (ribs 6–9) and hemorrhage to MAP 40 ± 5 mmHg for 2 h followed by crystalloid/autologous blood resuscitation	Not stated
Pilarczyk et al. [25]	Saline lavage (1000 mL bilaterally at 37 °C) repeated every 60 mins until injury achieved	PaO_2_ < 100 mmHg for > 1 h
Park et al. [24]	Saline lavage (1000 mL at 37 °C) repeated until injury achieved and fecal peritonitis (1 g/kg injection of feces into peritoneal cavity)	P/F < 50 mmHg
Kopp et al. [20]	Hypoxia (FiO_2_ reduced to achieve hypoxic inspiratory gas mixture)	SaO_2_ < 85%
Song et al. [26]	18–20 μg/kg i.v. *E. coli* LPS within 1 h	P/F ≤ 300 mmHg and 30% decrease in dynamic compliance from baseline
Kopp et al. [21]	Saline lavage (40 mL/kg) repeated until injury achieved	P/F < 100 mmHg
Henderson et al. [16]	0.2 mL/kg i.v. OA over 30 mins	P/F < 125 mmHg or HR < 60 bpm and/or reduction MAP > 50% from baseline
Dembinski et al. [13]	Saline lavage (40 mL/kg at 37 °C) repeated until injury achieved	PaO_2_ < 100 mmHg for > 1 h
Kocyildrim et al. [19]	3.5 μg/kg i.v. *E. coli* LPS over 30 mins	Not stated
Hou et al. [17]	Hypoxia (discontinuation of mechanical ventilation)	Not stated
Langer et al. [22]	0.1–0.15 mL/kg i.v. OA	P/F < 200 mmHg
Shekar et al. [14]	Smoke inhalation (10–12 mL/kg Vt breaths of cotton smoke, first cycle 12 breaths, then cycles of 8 breaths) repeated until injury achieved	Carboxyhemaglobin 45–50%
Totapally et al. [27]	Saline lavage (mL/kg) repeated × 3 and 2.5 mL/kg i.t. 0.1 N HCL	Not stated
Germann et al. [15]	0.5 mL/kg i.v. OA over 15 mins	LIS > 2.5

*OA* oleic acid, *i.v.* intravenous, *PEEP* positive end expiratory pressure, *FiO2* inspired fraction of oxygen, *P/F* ratio of arterial partial pressure of oxygen to inspired fraction of oxygen, *LPS* lipopolysaccharide, *MAP* mean arterial pressure, *PaO2* arterial partial pressure of oxygen, *bpm* beats per minute, *Vt* tidal volume, *HCL* hydrochloric acid

**Table 4:** Details of ventilatory management before and during ECMO

Study	Ventilator strategy										Gas exchange targets					
	Before ECMO					During ECMO					Before ECMO			During ECMO		
	Mode	Vt	PEEP	RR	FiO_2_	Mode	Vt	PEEP	RR	FiO_2_	PaO_2_	SpO_2_	PaCO_2_	PaO_2_	SpO_2_	PaCO_2_
		mL/kg	cmH_2_O	b/min			mL/kg	cmH_2_O	b/min		mmHg		mmHg	mmHg		mmHg
Dogs																
Kim et al. [18]					0.4						150–250		35–45			
Pigs																
Araos et al. [12]	VC	10	5	16–18	1.0	VC	10	5	nPaCO_2_				35–50			30–50
Wang et al. [28]	PC	7–9	0	30	0.21–0.30	PC	7–9	2–4	20–25	0.3–0.5						35–45
Ni et al. [23]						VC	8	5	15	0.5						
Pilarczyk et al. [25]	PC	6	5	15	1.0											
Park et al. [24]		8	5	nPaCO_2_	1.0			VAR				94–96	35–45	VAR	VAR	VAR
Kopp et al. [20]				nPaCO_2_	1.0					0.2						
Song et al. [26]	PC	7–9	0	30	0.21–0.35	PC		2–4	10–30	0.21–0.5	> 60		35–45	> 60		35–45
Kopp et al. [21]		10	5	nPaCO_2_	1.0	PC	6–8	8		VAR			NORM	60–80		NORM
Henderson et al. [16]		10–15	5	10	0.4								NORM	200–300		35–45
Dembinski et al. [13]	VC	8	5	nPaCO_2_	1.0								NORM			
Sheep																
Kocyildrim et al. [19]		10		12–15	0.6		6–7	5	10–12	0.21			35–40			
Hou et al. [17]		6–8		16–18			6–8		16–18							
Langer et al. [22]	CPAP	VAR	8	VAR	0.5	CPAP	VAR	8	VAR	0.5						VAR
Shekar et al. [14]						VC	4–6	10	6	0.21						
Totapally et al. [27]		7	4	nPaCO_2_	1	CMV			VAR				35–45			35–45
Germann et al. [15]	PC		0–10		0.3–0.7						> 70					

*Vt* tidal volume, *PEEP* positive end expiratory pressure, *RR* respiratory rate, *FiO*_*2*_ inspired fraction of oxygen, *PaO*_*2*_ arterial partial pressure of oxygen, *SpO*_*2*_ peripheral oxygen saturation, *PaCO*_*2*_ arterial partial pressure of carbon dioxide, *nPaCO*_*2*_ to maintain PaCO_2_ in normal range, *VC* volume controlled, *PC* pressure controlled, *CPAP* continuous positive airway pressure, *NORM* to ‘normal range’, *VAR* varied

### Models of ECMO and its intra-experimental management

Most studies performed veno-venous ECMO (*n* = 13). A summary of ECMO models and the management of ECMO during experiments are provided in Table 5. In most cases (*n* = 14), cannulation was peripheral, with three studies performing surgical cutdown [17, 25, 28]. There were a wide variety of cannulation configurations among studies. Few studies described a means of confirming cannula positioning, although peripheral ultrasonography [17, 24], intracardiac sonography [14], and a pressure guided method [22, 29] were reported. A range of commercial and experimental pumps and oxygenators were used. The constituents of priming solutions were described in less than half of the studies (*n* = 8) but included saline [12, 13, 24], lactated Ringers (LR) [25], albumin and saline [16], hydroxyethyl starch (HES) and LR [21], Voluven and LR [23], and Plasmalyte-148 and albumin [14]. The use of heparin as an anticoagulant was ubiquitous.

**Table 5:** Details of ECMO management

Study	ECMO type		ECMO equipment			ECMO settings			Anticoagulation	
	Mode	Configuration	Pump	Oxygenator	Cannula size (Fr) A-R	Flow	Sweep gas	FiO_2_	Type	ACT target (s)
Dogs										
Kim et al. [18]	VAc	RA–Ao	Multiple	Multiple	23–19	1.2–2 L/min	1.8–2 L/min	0.6		
Pigs										
Araos et al. [12]	VV	EJV–EJV	Medtronic Bioconsole 540	Medos HILTE 2400LT	23 dual-lumen	65 mL/kg/min	65 mL/kg/min		Heparin	180–220
Wang et al. [28]	VV	EJV–FV	Maquet Jostra	Medos HILTE 2400LT	12–8	70–80 mL/kg/min		1.0	Heparin	180–220
Ni et al. [23]	VV	FV–IJV	Maquet Rotaflow	Maquet Quadrox D	14–14	50 mL/kg/min	50 mL/kg/min	1.0	Heparin	180–220
Pilarczyk et al. [25]	VV	FV–EJV	Multiple	Multiple	23–21	2.4–2.8 L/min	3 L/min		Heparin	180–220
Park et al. [24]	VV	FV - EVJ	Maquet Rotaflow	Maquet Quadrox D	20/21–20/21	0.5–3 L/min	2:1–1:2 BF:GF		Heparin	1.5–2.5 × baseline
Kopp et al. [20]	VV	FV–EJV	Experimental	Experimental	19–17	30–40% CO	2 L/min		Heparin	≥ 149
Song et al. [26]	VV	EVJ–FV	Maquet Jostra	Medos HILTE 2400LT	14–12	70–80 mL/kg/min	2 L/min	1.0	Heparin	180–220
Kopp et al. [21]	VV	FV – EVJ	Multiple	Multiple	Multiple	25–40% CO	3–6 L/min		Heparin	120–150
Henderson et al. [16]	VA	EJV–CA	Stockert roller pump		8–10	100 mL/kg/min			Heparin	180–220
Dembinski et al. [13]	VV	FV–FV	Medos DeltaStream	Medos HILTE 7000	17–15	30% CO	30% CO	1.0	Heparin	≥ 130
Sheep										
Kocyildrim et al. [19]	VV	SVC–PA	Thoratec Centrimag	Xenios iLA	24–24	1.2–1.4 L/min			Heparin	> 200
Hou et al. [17]	VAc	Multiple	Maquet Rotaflow	Maquet Quadrox D	19–15	50 mL/kg/min	50 mL/kg/min	1.0	Heparin	180–220
Langer et al. [22]	VV	EJV–EJV	Maquet Cardiohelp	Maquet HLS Set	23 dual-lumen	2 L/min	1–10 L/min	0.5–1.0	Heparin	> 160
Shekar et al. [14]	VV	EJV–EJV	Maquet Rotaflow	Maquet Quadrox D	21–19	60–80 mL/kg/min	80% pump flow	1.0	Heparin	220–250
Totapally et al. [27]	VA	IJV–CA		Medtronic Minimax		15% CO	1 L/min	1.0	Heparin	
Germann et al. [15]	VVc	IVC–SVC	Stockert roller pump	Medtronic Maxima+		2.5–3.5 L/min		0.21–1.0		

*Fr* French, *FiO2* inspired fraction of oxygen, *A–R* access–return, *ACT* activated clotting time, *Vac* central veno-arterial, *RA* right atrium, *Ao* aorta, *VV* veno-venous, *EJV* external jugular vein, *FV* femoral vein, *IJV* internal jugular vein, *VA* veno-arterial, *CA* carotid artery, *SVC* superior vena cava, *PA* pulmonary artery, *VVc* central veno-veno

### ARRIVE compliance

No study published after 2011 explicitly referenced the ARRIVE standards or reported compliance with them.

## Discussion

This systematic review provides the first detailed overview of animal models which combine features of experimental ARDS with ECMO. In doing so, we have demonstrated marked heterogeneity in both their design and reporting.

Animal models play a key role in research into ARDS and are well established in both small [30] and large animal species [31]. Given the complexity of the underlying pathophysiology, they are essential tools for deriving new mechanistic insights as well as establishing the efficacy and safety of novel interventions [32]. Their place in current ECMO research is less clear. Our study found no example of a contemporary small animal model combining features of ARDS and ECMO. This may reflect the inherent difficulties of replicating a clinically relevant extracorporeal circulation in a small animal species, although such models have been described in the absence of lung injury in rodents [33] and rabbits [34, 35]. While small animal models are limited by the inability to use clinical ECMO devices, differences in lung morphology [36], and variations in innate immunity [37], they offer several advantages. Studies involving small animal species are less resource intensive than those in large animals, can be conducted more quickly, may take advantage of varied genetic strains, and have the advantage of using multiple assays and imaging techniques not available in large animals.

All models identified by our study were conducted in large animals. These models may have advantages, which are generally the converse of the limitations seen in small animals. A feature of studies in our review is their relatively short duration, with only two models describing recovery and follow-up beyond 24 h [26]. This may be a result of the intensive and costly nature of large animal studies, although models supported for more than 24 h and/or those with the potential for recovery would be of benefit in addressing important research questions. In the context of ARDS and ECMO, models of greater duration would facilitate research into the proliferative phase of lung injury, allow investigators to explore lung recovery during ECMO, and could test approaches to weaning from extracorporeal support.

Regardless of species, models of experimental ARDS identified in this study were diverse. Previously, the American Thoracic Society (ATS) has attempted to standardize experimental ARDS by identifying core pathophysiological features which should be established in pre-clinical models [38]. In our review, few studies published after the ATS workshop report acknowledge these features or reported compliance. To increase the validity of studies, the presence or absence of these features should be evaluated during model development. Most commonly described means of inducing lung injury were described: saline lavage, oleic acid infusion, endotoxemia, acid aspiration, and smoke inhalation. Notably, we failed to identify a study which included the use of live bacteria, a method frequently employed in singular models of experimental ARDS [31]. Recent work, using latent class analysis (LCA), has identified stable ARDS phenotypes present in large clinical trial cohorts. These have been broadly represented as ‘hyper-’ or ‘hypo-inflammatory’, each group having distinct clinical and biological features. Importantly, sub-phenotypes also appear to have differing responses to treatment and variations in outcome [39–41]. This work has implications for the design of pre-clinical studies. In our review, there is a preponderance toward models which likely induce ‘hypo-inflammatory’ ARDS, such as oleic acid infusion and saline lavage, both have which have been associated with a failure to induce pro-inflammatory cytokines or significant neutrophil influx to the lung [31]. In future, investigators should consider phenotypes when contemplating a method of injury. Regardless of the method of achieving experimental ARDS in animals, models that incorporate ECMO must also take account of the severity of the disease. Only four studies identified by our review targeted an injury which delivered a partial pressure of oxygen to inspired fraction of oxygen (P/F) ratio of less than 100 mmHg [13, 20, 24, 25]. No included study evaluated ventilatory pressures or the presence of acidosis as part of the definition of injury. Future models, particularly those used to assess interventions during ECMO, should aim to replicate clinically meaningful injury criteria such as those used for inclusion into large clinical trials [3].

The supportive care administered to animals in included studies was an area of significant variation. The choice of agent for the induction and maintenance of anesthesia differed between studies, although almost all employed a total intravenous approach. The influence of anesthesia on outcomes of interest should be considered during the design of a model, and this is particularly true in respect of inhalational agents where emerging evidence points toward a potential role in modifying the inflammatory response associated with ARDS [42]. Reassuringly, most models described combining anesthetic and analgesic infusions, commonly with the addition of fentanyl. Only four models reported the use of neuromuscular blockade [12, 17, 24, 27]. Ten of the models were reported after publication of the ACURASYS study, which reported an improvement in mortality among patients with severe ARDS receiving early paralysis [43]. While some models may seek to evaluate spontaneous breathing during ECMO, neuromuscular blockade should be considered a standard of care in severe ARDS and thus be replicated as a feature of a high fidelity pre-clinical model.

Mechanical ventilation practices, both before and after the institution of ECMO, were poorly described. Few studies instituted lung-protective ventilation prior to ECMO and many described using tidal volumes in excess of 8 mL/kg. Given the clear evidence for low tidal volume ventilation in ARDS [44], failure to implement this in pre-clinical models limits their validity. While the evidence supporting approaches to ventilation during ECMO is less well defined, only one model reported the use of an ultra-protective ventilatory strategy [14]. Levels of positive end-expiratory pressure (PEEP) during ECMO also appear low when compared with contemporary clinical practice [45]. Considering the importance of mechanical ventilation in ARDS and its ability to aggravate injury through ventilator-induced lung injury (VILI), models of ARDS and ECMO should at a minimum provide a detailed description of ventilatory practices.

In general, reporting of ECMO was more complete. All models provided a description of cannula configuration. In the future, investigators should use the Extracorporeal Life Support Organization Maastricht Treaty on ECMO nomenclature to ensure consistency and clarity [46]. As would be expected, models employed a variety of ECMO devices, many of which are in contemporary clinical use. While flow and sweep gas settings were well reported, few studies provided details on gas exchange targets during ECMO, with only 1 in 4 stating a target PaO_2_ and less than half providing a target PaCO_2_. Heparin was the anticoagulant of choice in every model that provided details of anticoagulation practice. Likewise, all but three studies provided target activated clotting time (ACT) ranges. The ubiquity of ACT may reflect the relatively short duration of included models and the requirement for a cost-effective bedside measure of coagulation. Anticoagulation targets varied between models, which may reflect continuing uncertainty as to the optimal clinical regime [47].

No study identified by this review, and published after 2011, explicitly referenced the ARRIVE guidelines for improving the reporting of animal studies [11]. This is perhaps not a feature limited to models of ARDS and ECMO, but instead reflects a wider issue with adherence despite widespread support for the standard [48]. While adherence to the ARRIVE standards (or similar) is likely to enhance the quality and reproducibility of published studies, there are many subject-specific domains (e.g., technical aspects of ECMO, mechanical ventilation practices) which are equally important but omitted by these higher-level guidelines. Several initiatives have attempted to address this in pre-clinical stroke models and more recently in sepsis. Here we have outlined what domains a minimum reporting standard for pre-clinical models of ARDS and ECMO may contain (Table 6).

**Table 6:** Proposed domains of a minimum reporting standard for pre-clinical studies of ARDS and ECMO

Domains	Example items	Notes
1. ARDS model and definition	Method of injury, including dosing and duration	Should be consistent with ATS report [38]
	Description of validation	
	Operational definition of injury	
2. Mechanical ventilation	Mode of ventilation	
	Target tidal volume	
	PEEP settings	
	Ventilatory strategy during ECMO	
3. Supportive care	Use of neuromuscular blockade	
	Prone positioning	
	Fluid therapy–type and quantity	
4. ECMO equipment	Pump and oxygenator make and model	
	Cannulae make and model	
5. ECMO cannulation	Standard description of configuration	Should use Maastricht treaty nomenclature [46]
	Method of cannulation	
6. ECMO management	Flow targets	
	Gas exchange targets/sweep gas management	
	Anticoagulation strategy and targets	

### Limitations

Our review has several limitations. Firstly, despite each included study being the first description of a combined model of ARDS and ECMO, occasionally investigators used components of previous instances of experimental ARDS or ECMO in creating them. Where such studies were referenced, we made every attempt to retrieve relevant data. Secondly, no formal risk of bias assessment was undertaken as part of this review. While this limited our ability to assess the quality of included studies, the principal aim of our review was to identify and describe models. Finally, an arbitrary date was used to exclude historical models of ARDS and ECMO. This was pre-judged to allow consideration of models most likely to have contemporary clinical significance but may have excluded older models which remain viable.

## Conclusion

A limited number of models combine the features of experimental ARDS with ECMO. Among those that exist, there is significant heterogeneity in both design and reporting. This creates difficulty in assessing results and in generalizing findings to clinical settings. There is a need to standardize the reporting of pre-clinical studies using in this area. This could be achieved by the introduction of a minimum data set for pre-clinical ECMO studies.

## Additional files

Additional file 1: The search strategy designed in conjunction with a trained medical librarian (DOCX 20 kb)

Additional file 2: Animal models of ECMO and ARDS systematic review data extraction (DOCX 16 kb)

## Abbreviations

ACT: Activated clotting time
Ao: Aorta
ARDS: Acute respiratory distress syndrome
Bpm: Beats per minute
CA: Carotid artery
CMV: Continuous mandatory ventilation
ECMO: Extracorporeal membrane oxygenation
EJV: External jugular vein
ETT: Endotracheal tube
FA: Femoral artery
FiO_2_: Inspired fraction of oxygen
Fr: French
FV: Femoral vein
HCL: Hydrochloric acid
HES: Hydroxyethyl starch
IJV: Internal jugular vein
LPS: Lipopolysaccharide
LR: Lactated Ringers
MAP: Mean arterial pressure
OA: Oleic acid
P/F: Ratio of arterial partial pressure of oxygen to inspired fraction of oxygen
PA: Pulmonary artery
PaCO_2_: Arterial partial pressure of carbon dioxide
PaO_2_: Arterial partial pressure of oxygen
PC: Pressure controlled
PEEP: Positive end-expiratory pressure
Rpm: Revolutions per minute
SpO_2_: Peripheral oxygen saturation
SVC: Superior vena cava
Trach: Tracheostomy
VA: veno-arterial
VC: Volume controlled
Vt: Tidal volume
VV: Veno-venous
