# Supplementary material for: Extracorporeal membrane oxygenation (ECMO) and the acute respiratory distress syndrome (ARDS): a systematic review of pre-clinical models
Source: Intensive Care Med Exp. 2019 Mar 25;7:18. doi: 10.1186/s40635-019-0232-7 (PMC6434011; doi:10.1186/s40635-019-0232-7)
Supplement: Supplementary file 2 — Animal models of ECMO and ARDS systematic review data extraction (DOCX 16 kb) [file 40635_2019_232_MOESM2_ESM.docx]

**Supplementary File 2.**

Animal Models of ECMO and ARDS Systematic Review Data Extraction

| **STUDY** | |
| --- | --- |
| Title |  |
| 1^st^ Author |  |
| Year |  |
| Journal |  |
| **ANIMALS** | |
| Animal Species |  |
| Age |  |
| Weight |  |
| Gender |  |
| Total number used |  |
| Subgroups |  |
| **STUDY** | |
| Intervention |  |
| Duration |  |
| **ANAESTHESIA** | |
| Induction |  |
| Maintenance |  |
| Airway |  |
| Ventilation |  |
| Monitoring |  |
| Study Drugs or Treatments |  |
| Euthanasia |  |
| **ALI/ARDS Model** | |
| Definition |  |
| Method |  |
| Time to injury |  |
| Previously described |  |
| **ECMO** | |
| Mode |  |
| Cannulation |  |
| Flow |  |
| FiO_2_ |  |
| PaCO_2_ |  |
| Anticoagulation |  |
| ACT Target |  |
| ECMO duration |  |
| ECMO timing |  |
| Complications |  |
| Mortality |  |
